# Antimicrobial susceptibility patterns of Escherichia coli from various clinical sources

**DOI:** 10.1017/ash.2025.177

**Published:** 2025-08-15

**Authors:** Waleed A. Al Qahtani, Mohamed S. Zaghlol, Yazeed Ali Mohammed Qasem, Mohsen M. Mashi, Ahmed A. Daghriri, Yahya kubaysi, Hisham N. Hakami, Alallah F. Khawaji, Nabil T. Hakami, Mohammed A. Jeraiby, Hassan N. Moafa, Mohammed Ezzi, Jobran M Moshi

**Affiliations:** 1 Laboratory Department, Armed Force Hospital, Jazan, Kingdom of Saudi Arabia; 2 Department of Clinical Pathology, Faculty of Medicine, Al-Azhar University, Assiut, Egypt; 3 Basic Medical Science, faculty of medicine, Jazan University, Jazan, Kingdom of Saudi Arabia; 4 Department of Public Health, College of Nursing and Health Sciences, Jazan University, Jazan, Kingdom of Saudi Arabia; 5 Department of Surgery, Faculty of Medicine, Jazan University, Jazan, Kingdom of Saudi Arabia; 6 Department of Medical Laboratory Technology, College of Nursing and Health Science, Jazan University, Jazan, Kingdom of Saudi Arabia. Health Research Centre, Jazan University, Jazan, Kingdom of Saudi Arabia

## Abstract

**Background:**

*Escherichia coli*, frequently abbreviated as *E. coli*, is a common gastrointestinal tract inhabitant in both people and animals. It may also be found in soil, aquatic settings, and plants.

**Aim:**

The purpose of the study was to identify the frequency and three susceptibilities of *E. coli* in various clinical samples taken from patients in the Jazan area.

**Materials and methods:**

Using a multi-center approach, this retrospective cross-sectional study analyzed the results of culture and antimicrobial susceptibility of isolates from urine, wound swabs, and sputum samples. The study covered the period from January 2023 to December 2023 and included all public and private hospitals in the Jazan region.

**Results:**

The majority of isolates were derived from urine samples in 1161 patients (85.49%), followed by pus in 123 patients (9.06%) and sputum in 74 patients (5.45%). There were high sensitivity rates to Amikacin, Tigecycline, and Imipenem by (97.49%), (90.87%), and (90.35%), respectively, while there were high resistance rates to Norfloxacin, Ampicillin, and Cefotaxime by (93.67%), (79.60%), and (71.65%), respectively.

**Conclusion:**

There was considerable resistance to commonly used antibiotics among *Escherichia coli* germs isolated from several clinical specimens. Antibiotics, including imipenem, amikacin, and nitrofurantoin, demonstrated the highest efficacy against *E. coli* isolates. Nalidixic acid, cefexime, and ceftriaxone showed efficacy against *E. coli*; nevertheless, several clinical isolates exhibited resistance.

## Introduction

The bacterium Escherichia coli, more often known as *E. coli*, is common in the human and animal digestive systems as well as in the environment, including plants, water, and soil.^1^ Among human infections, wounds, otitis media, and bloodstream infections, it ranks first among these pathogens and is the most prevalent cause of UTIs.^2^ In underdeveloped nations, *E. coli* is the leading etiology of water & food-borne diarrhea in humans, and it kills a lot of kids younger than five.^3^

*E. coli* has been shown to be resistant to several antibiotics, and this is becoming an increasingly serious problem in both industrialized and poor nations. Bacterial resistance to antibiotics is on the rise, which makes illness treatment more difficult.^4^ It is common practice to treat people with severe symptoms without doing bacteriological investigations in as many as 95% of instances.^5^

The frequency and susceptibility profiles of *Escherichia coli* can vary greatly depending on factors such as geography, demographics, and the environment.

This study intended to ascertain the frequency and antibiotic susceptibility of *E. coli* from various clinical samples in the Jazan area.

## Materials and methods

The antibiotic susceptibility and culture findings of isolates from sputum, wound swabs, and urine were examined in this retrospective cross-sectional study. It was conducted through a multi-center approach involving various healthcare facilities in the Jazan Region, ensuring representative sampling from January 2023 to December 31, 2023, across all public and private hospitals in the area.

Inclusion criteria: All clinical samples positive for E. coli obtained from patients in the sharing centers in Jazan Region. Clinical sources include urine, blood, wound swabs, sputum, and other relevant specimens.

## Methods

### All participants were subjected to the following

#### Laboratory procedures

As part of standard operating procedure, samples were collected in sterile containers to ensure aseptic collection. The clean-catch midstream urine sample was collected in the morning using sterile wide-mouth glass containers. Calibrated wire loops were used to inoculate urine samples onto cystine lactose electrolyte-deficient medium, MacConkey agar, and blood agar (HiMedia Laboratories Pvt. Limited, India). After that, the samples were aerobically incubated at 37 ºC for 24 h. Positive cultures were analyzed using standard microbiological methods to identify uropathogens. When a urine culture yielded more than 105 colony-forming units/ml of urine, it was considered significant bacteriuria.^12^ Using sterile cotton swabs, pus was recovered from the wound. Sterile, wide-mouthed containers were used to collect sputum samples. Plates of MacConkey agar, blood agar, and chocolate agar were infected with specimens (HiMedia, India). After 24 and 48 hours of incubation at 37ºC in an aerobic environment, the plates were evaluated.

#### Microscopic examination

Gramme staining and careful examination were used to identify colonies that had been obtained from blood agar or Mac Conkey’s agar plates.

#### Antimicrobial susceptibility tests

Following standard operating procedure, Mueller-Hinton agar was subjected to antimicrobial susceptibility testing using the Kirby-Bauer disc diffusion technique. Automated procedures with BD Phoenix and Vitck equipment. The National Committee for Clinical Laboratory Standards’ standards were followed for analyzing the resistance data. As a quality control measure, antimicrobial susceptibility testing made use of *E. coli* ATCC 25922 and *S. aureus* ATCC 25923 reference strains. Traceability and confirmation of *Escherichia coli* by use of time-tested microbiological techniques. The Kirby-Bauer disc diffusion technique or automated systems performed antimicrobial susceptibility testing according to Clinical and Laboratory Standards Institute (CLSI) guidelines. Ampicillin, Ciprofloxacin, Trimethoprim/sulfamethoxazole, Ceftriaxone, and Meropenem are among the often prescribed antibiotics that should be included in the testing. The inhibitory zone width was measured to the nearest millimeter after a 24-hour incubation at 37°C. Isolates were classified as susceptible, intermediate, or resistant according to the criteria set by the Clinical and Laboratory Standards Institute. Standards offered by CLSI for interpretation.^6^

Data collection: Demographic data (age, gender) & clinical information (source of specimen, hospital ward) recorded for each patient. Antimicrobial susceptibility results are documented systematically, including zone diameters and interpretation.

#### Data analysis

A descriptive study was conducted to ascertain the distribution and frequency of *E. coli* isolates from various clinical sources. Calculation of antimicrobial susceptibility rates for each antibiotic tested. Statistical analysis to explore associations between clinical variables and antimicrobial resistance patterns (eg, chi-square test, logistic regression).

#### Ethical considerations

Protection of patient confidentiality and privacy throughout the study. Informed consent was acquired from patients or parents before sample collection. Adherence to ethical standards in research involving human subjects. The reasearch approved by Jazan Armed Force Hospital Ethical committee.

#### Quality control

Standardization of laboratory procedures and adherence to quality control measures to ensure accuracy and reliability of results. Regular calibration and quality assurance of laboratory equipment and antimicrobial disks.

## Results

*Escherichia coli* is among the most common bacteria that can cause illness.^7^ Severe health complications, including extended hospital stays and treatment failures, are caused by *E. coli* antibiotic resistance patterns, which continue to be a major public health concern globally.^8^ *E. coli* prevalence and antibiotic susceptibility from clinical samples in Jazan were the focus of this study.

**Figure 1.**
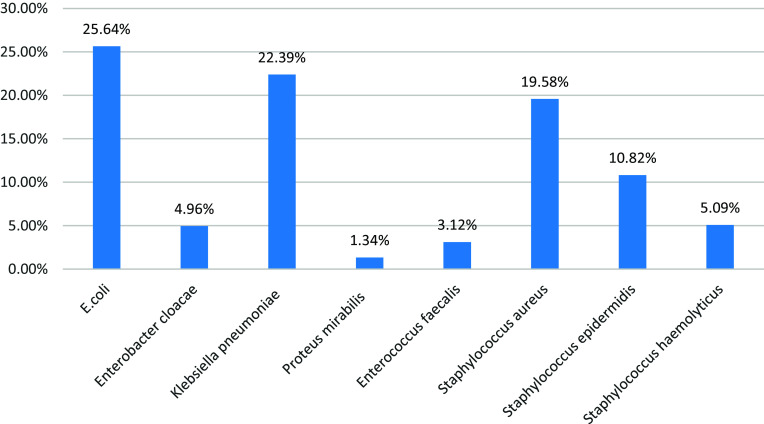
Shows distribution of positive culture isolates.

**Figure 2.**
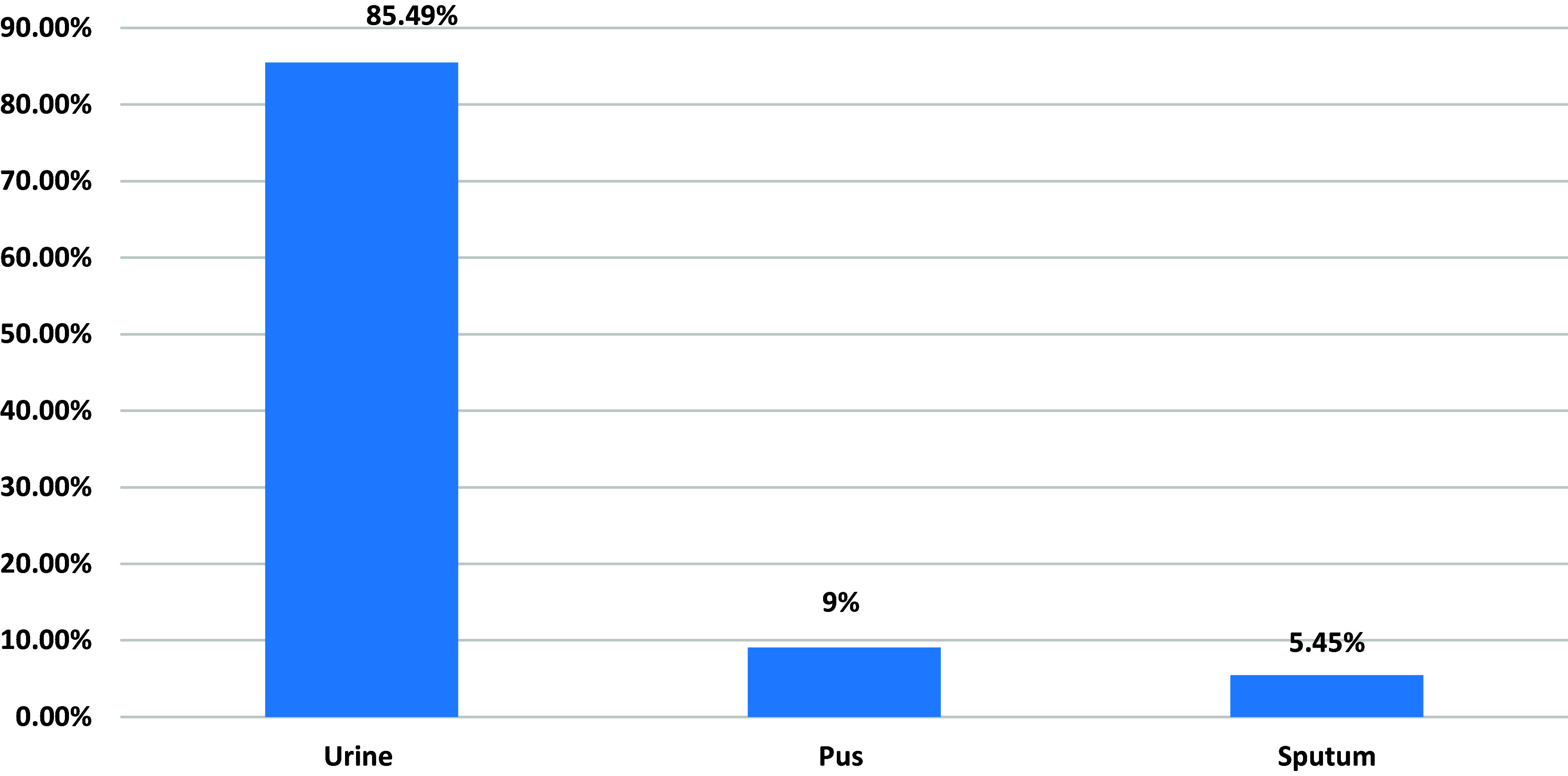
Shows distribution of specimen in studied group.

**Figure 3.**
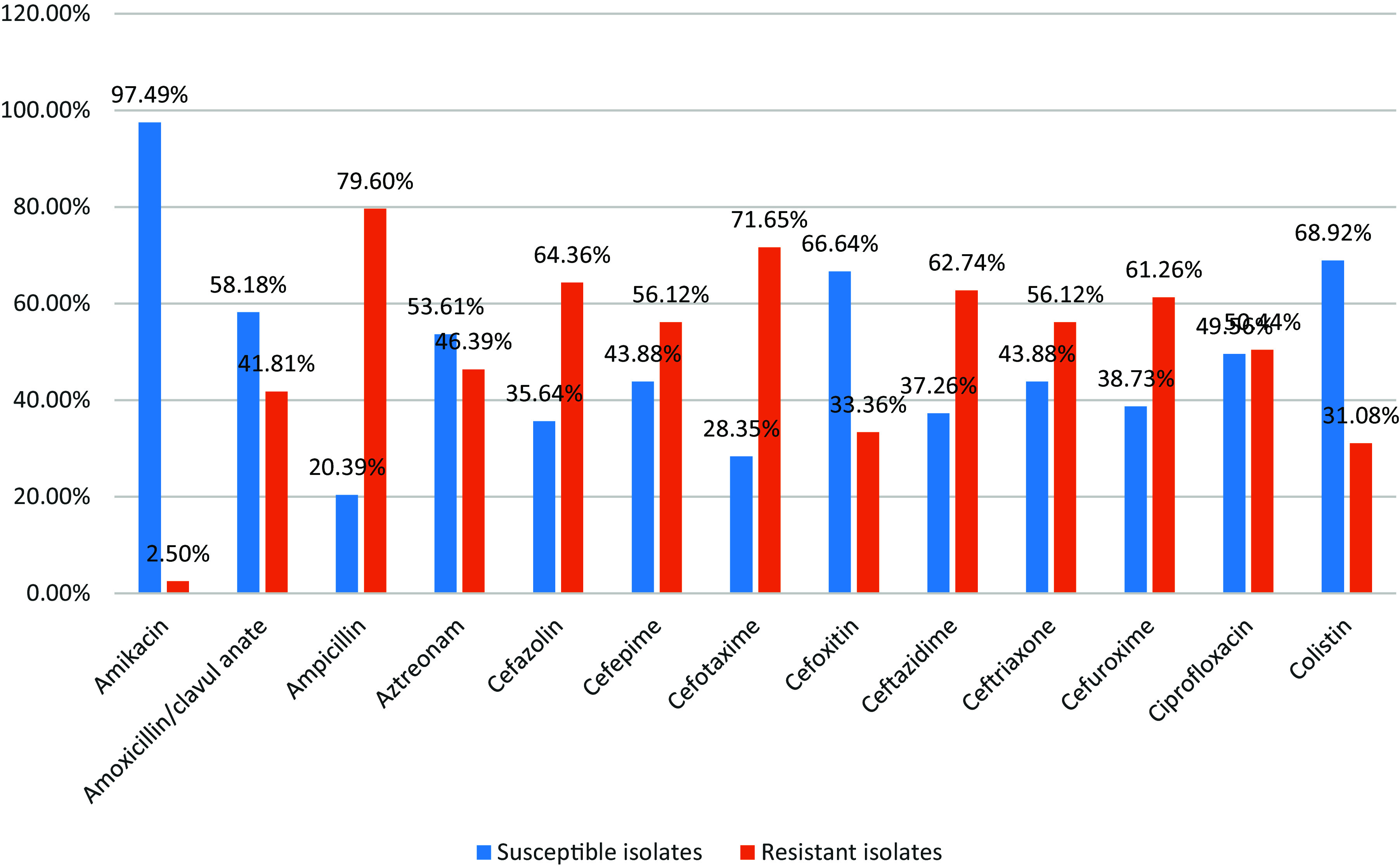
Shows distribution of antimicrobial susceptibility pattern of *E. coli* isolates.

**Figure 4.**
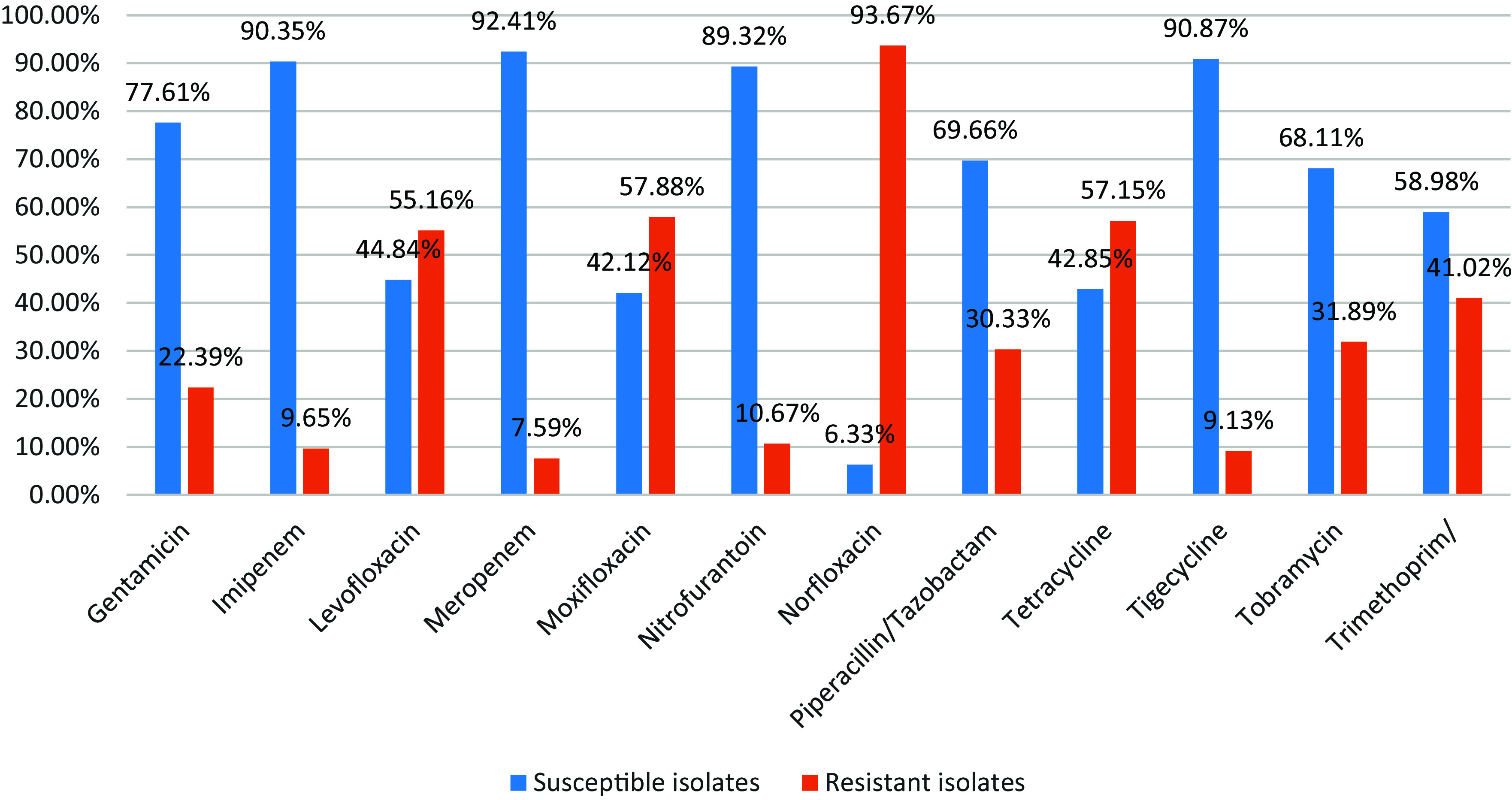
Shows distribution of antimicrobial susceptibility pattern of *E. coli* isolates.

This study reported that 41 patients (20.56%) their age was less than 20, 67 patients (33.57%) their age ranged from 20 to 40, 92 patients (45.88%) their age ranged from 40 to 60, 53 patients (26.51%) were males, and 147 patients (73.49%) were females.

In addition, Naqid et al.^9^ identified the sensitivity pattern of Escherichia coli bacteria obtained from several clinical sources in Duhok city, Iraq. A total of 454 samples were collected, with 321 women and 133 males included. The age range of the participants was ten to sixty. Urine samples from females were found to have higher clinical isolates of Escherichia coli compared to male samples.

Consistent with prior studies, this finding shows that females are more likely to get UTIs caused by *E. coli*.^10,11^

The reason this microbe is more common in girls than males is because germs can’t travel as far via their shorter urethras to reach the bladder and infect the bladder more easily during sexual activities.^12^ Low socioeconomic position and inadequate personal hygiene exacerbate these risk factors for UTI.^13^

Our results showed that 25.64% of patients had positive culture *E. coli*, the majority of isolates were obtained from the urine samples in 171 patients (85.49%), followed by pus in 18 patients (9.06%), and sputum in 11 patients (5.45%).

The findings of this study were in line with those of Naqid et al.^9^ who reported that the majority of the E. coli isolates were obtained from urine samples (418; 92.2%). This was followed by wound samples (18; 3.9%), cervical samples (7; 1.5%), blood samples (4; 0.9%), semen samples (3; 0.7%), ascitic samples (2; 0.4%), and cerebral spinal fluid samples (2; 0.4%).

As well, the present study agreed with Eltabey et al.,^14^ who reported that the common isolates were obtained from the urine samples.

Our findings demonstrated that there were high sensitivity rates to Amikacin, Tigecycline and Imipenem by (97.49%), (90.87%) and (90.35%) respectively, while there were high resistance rates to Norfloxacin, Ampicillin and Cefotaxime by (93.67%), (79.60%) and (71.65%) respectively.

The results were in agreement with those of Naqid et al., who had previously shown that 88.3% of the *E. coli* strains they had identified were resistant to ampicillin, 63.9% to ceftriaxone, and 63.9% to cefepime. However, it was shown that *E. coli* was quite sensitive.^9^

Consistent with other studies, we found that imipenem had a profound effect on *E. coli* isolates obtained from urine.^16^

Odongo et al. also determined that Escherichia coli isolates were highly susceptible to cefotaxime/clavulanic acid (100%) and nitrofurantoin (70%), but very resistant to cefuroxime (100%), ceftazidime (100%), nalidixic acid (90%), and ciprofloxacin (90%). The results of this study were in agreement with those of Odongo et al.^17^

Furthermore, according to Kasanga et al. the majority of the isolates tested positive for resistance to ampicillin (81.4%), sulfamethoxazole/trimethoprim (70.7%), ciprofloxacin (67.9%), levofloxacin (64.6%), ceftriaxone (62.3%), and cefuroxime (62%), Yet, amikacin (100%), imipenem (99.5%), nitrofurantoin (89.3%), ceftolozane/tazobactam (82%), and gentamicin (72.1%) were all extremely effective against *E. coli* isolates.^18^

Eltabey et al. also discovered that almost all of the tested isolates were responsive to Amikacin (90%) and Meropenem (92%); however, all of the isolates were resistant to Amoxicillin/clavulanic acid (100%) and Cefotaxime (44%).^14^

**Table 1.** Distribution of positive culture isolates in all studied patients

	Total positive culture N = 5295	
	N	%
*E. coli*	1358	25.64%
*Enterobacter cloacae*	263	4.96%
*Klebsiella pneumoniae*	1186	22.39%
*Proteus mirabilis*	71	1.34%
*Enterococcus faecalis*	165	3.12%
*Staphylococcus aureus*	1037	19.58%
*Staphylococcus epidermidis*	573	10.82%
*Staphylococcus haemolyticus*	270	5.09%

This table shows that 25.64% of patients had positive culture *E. coli*, 22.39% of patients had positive culture *Klebsiella pneumoniae*, 19.58% of patients had positive culture *Staphylococcus aureus*, 10.82% of cases had +ve culture *Staphylococcus epidermidis*, 5.09% of patients had positive culture *Staphylococcus haemolyticus*, 4.96% of patients had positive culture *Enterobacter cloacae*, 3.12% of patients had positive culture *Enterococcus faecalis* and 1.34% of patients had positive culture *Proteus mirabilis* (Table 1).

**Table 2.** Distribution of demographic data in patients with positive culture *E. coli*

	Positive culture *E. coli* N = 1358	
	N	%
**Age**		
<20	279	20.56%
20–40	456	33.57%
40–60	623	45.88%
**Sex**		
Male	360	26.51%
Female	998	73.49%

This table shows that 279 patients (20.56%) their age <20, 456 patients (33.57%) their age ranged from 20 to 40, 623 patients (45.88%) their age ranged from 40 to 60, 360 patients (26.51%) were males, and 998 patients (73.49 %) were females (Table 2).

**Table 3.** Distribution of specimens in patients with positive culture of *E. coli*

	Positive culture *E. coli* N = 1358	
	N	%
Urine	1161	85.49%
Pus	123	9.06%
Sputum	74	5.45%

The majority of isolates were obtained from the urine samples in 1161 patients (85.49%), followed by pus in 123 patients (9.06%) and sputum in 74 patients (5.45%) (Table 3).

**Table 4.** *E. coli* isolates’ responsiveness to various antibiotics

	Positive culture *E. coli* N = 1358			
	Susceptible isolates		Resistant isolates	
	N	%	N	%
Amikacin	1324	97.49%	34	2.50%
Amoxicillin/clavulanate	732	58.18%	626	41.81%
Ampicillin	277	20.39%	1081	79.60%
Aztreonam	728	53.61%	630	46.39%
Cefazolin	484	35.64%	874	64.36%
Cefepime	596	43.88%	762	56.12%
Cefotaxime	385	28.35%	973	71.65%
Cefoxitin	905	66.64%	453	33.36%
Ceftazidime	506	37.26%	852	62.74%
Ceftriaxone	596	43.88%	762	56.12%
Cefuroxime	526	38.73%	832	61.26%
Ciprofloxacin	673	49.56%	685	50.44%
Colistin	936	68.92%	422	31.08%
Gentamicin	1054	77.61%	304	22.39%
Imipenem	1227	90.35%	131	9.65%
Levofloxacin	609	44.84%	749	55.16%
Meropenem	1255	92.41%	103	7.59%
Moxifloxacin	572	42.12%	786	57.88%
Nitrofurantoin	1213	89.32%	145	10.67%
Norfloxacin	86	6.33%	1272	93.67%
Piperacillin/Tazobactam	946	69.66%	412	30.33
Tetracycline	582	42.85%	776	57.15%
Tigecycline	1234	90.87%	124	9.13%
Tobramycin	925	68.11%	433	31.89%
Trimethoprim/ sulfamethoxazole	801	58.98%	557	41.02%

There were high sensitivity rates to Amikacin, Tigecycline, and Imipenem by (97.49%), (90.87%), and (90.35%), respectively, while there were high resistance rates to Norfloxacin, Ampicillin & Cefotaxime by (93.67%), (79.60%), and (71.65%) respectively (Table 4).

## Conclusion

*Escherichia coli* bacteria isolated from different types of human tissues showed distinct antibiotic sensitivity patterns. A considerable degree of resistance to commonly used antibiotics was demonstrated by these microorganisms. Of the medicines tested, amikacin, tigecycline, and imipenem were the most effective against the different strains of *E. coli*. Instead, the clinical isolates of *E. coli* exhibited increased levels of resistance to the antibiotics norfloxacin, ampicillin, and cefotaxime. As a result, it is suggested that antibiotic sensitivity testing be carried out by medical professionals in order to choose the antibiotics that are the most effective.
